# A research on spatial configuration characteristics and social performance evaluation of public sports facilities in shanghai based on geographic information system (GIS)

**DOI:** 10.1371/journal.pone.0310585

**Published:** 2025-05-12

**Authors:** Haonan Li, Lun Li, Yuan Li, Qing Ji, Jianbo Zhao, Zixi Ge, Qi Zhou, Quan Sun

**Affiliations:** 1 School of Physical Education, China University of Geosciences (Wuhan), Wuhan, China; 2 School of Geography and Information Engineering, China University of Geosciences (Wuhan), Wuhan, China; King Abdulaziz University, SAUDI ARABIA

## Abstract

The spatial configuration and social performance of public sports facilities serve as crucial indicators for evaluating the equity of public sports services and the coherence of urban spatial structure. As Shanghai accelerates its development into a globally renowned sports city, the construction of public sports facilities has encountered significant opportunities. However, challenges persist in the spatial distribution, accessibility, and quality of these facilities. This study investigates the spatial agglomeration characteristics, accessibility, and social performance of urban public sports facilities in Shanghai at both the street and grid scales. Using geographic information system (GIS) tools and analytical methods such as kernel density estimation, standard deviation ellipse, spatial autocorrelation, Gaussian two-step moving search, and the Gini coefficient, the analysis yields the following findings: 1) Public sports facilities in Shanghai are concentrated in the central urban areas and exhibit scattered spatial distribution patterns in peripheral regions. These facilities display a significant directional coupling with population distribution (northeast-southwest), reflecting pronounced spatial imbalances. 2) Social performance analysis reveals clear regional inequities in Shanghai’s public sports facilities. While overall accessibility is relatively high, disparities remain, with suburbs facing facility shortages. Regional equity measurements indicate that the Gini coefficient for public sports facilities in Shanghai is 0.58. Central urban areas possess a high density of facilities, while suburban areas suffer from inadequate facility coverage, leading to uneven service radii and a pattern of high agglomeration but low coverage. 3) The social equity analysis shows that the service capacity entropy of public sports facilities exhibits a distinct spatial distribution, characterized by high values in the east and west and low values in the center. The highest entropy value is 4.25, while the lowest is 0.02. This study provides valuable insights for the planning and optimization of urban public sports facilities in Shanghai, contributing to the enhancement of spatial equity and service effectiveness.

## 1. Introduction

The report of the 20th Party Congress emphasizes the need to widely promote national fitness activities, encourage youth participation in sports, advance the comprehensive development of mass and competitive sports, and accelerate the construction of a sports powerhouse. Public sports facilities play a critical role in realizing citizens’ right to engage in sports. They are a key component of the public service system for national fitness, an essential guarantee for building a strong sports nation, and a foundational platform and driving force for fostering widespread fitness activities [1]. Strengthening the construction of public sports facilities and developing mass sports are vital public service responsibilities of governments at all levels. These efforts are also essential for implementing the national fitness strategy and the Healthy China initiative. As the core city of the world-class city cluster in the Yangtze River Delta and an international center for economics, finance, trade, shipping, and technological innovation, as well as a cultural hub, Shanghai has recognized the strategic importance of public sports facilities. In the “14th Five-Year Plan for the Construction of Public Sports Facilities in Shanghai” issued in December 2021, public sports facilities were identified not only as essential to Shanghai’s ambition to become a globally renowned sports city but also as prerequisites for achieving high-quality development. The plan calls for a comprehensive improvement in the layout of municipal, district, and community-level public sports facilities, the acceleration of facility supply, and the constant satisfaction of people’s growing demands for a better quality of life [2]. However, significant challenges remain. The current mismatch between public fitness demand and the supply of venues and facilities is pronounced, and the constraints imposed by a multi-factor austerity environment present a critical bottleneck that must be addressed. Academic research on the spatial configuration of public sports facilities can be broadly divided into three categories Firstly, research has focused on analyzing the service scope, spatial patterns, spatial evolution, and influencing factors of public sports facilities from various perspectives. For instance, Sun and Zhang investigated the spatial and temporal evolution of community sports and fitness venues in Shanghai from 1982 to 2019 using spatial analysis techniques. Their findings revealed a spatial evolution pattern characterized by the sequence of “urbanization-suburbanization-re-urbanization” [3]. Similarly, Huang and Gong conducted quantitative and qualitative analyses of outdoor sports venues in Nanchang City using spatial theory. They found that these venues followed a spatial distribution pattern of “sparse in the north and south, and strong agglomeration in the middle.” The COVID-19 pandemic caused the center of the standard deviation ellipse of these venues to shift southeastward, while the number of open-type venues significantly declined [4]. Additionally, studies on the spatial distribution of stadiums revealed patterns such as “more in the south, less in the north,” “dense in the southeast, sparse in the northwest,” and a belt-shaped distribution along coastal areas. The factors influencing these distributions show notable heterogeneity, including socio-economic and natural environmental factors [5].

Secondly, research has examined the factors affecting the spatial configuration of public sports facilities across different regions and countries. For example, Kruszyńska and Poczta analyzed the sports and recreation infrastructure in Poznań, identifying socio-economic, spatial-economic, and socio-spatial factors as key determinants of its spatial distribution [6].

Thirdly, studies have explored the connections between urban public fitness venues and broader topics such as urban public spaces, the COVID-19 pandemic, public health, urban green spaces, and social equity. Yi and Horton highlighted the underutilized potential of green spaces in China for fitness and exercise, emphasizing the need for effective integration of landscaped green belts, residential green spaces, and sports venues to maximize their value [7]. Liu linked urban public fitness venues to public health, illustrating the spatial disparities between the city center and fringe areas in Harbin, where the city center had more sports space resources compared to peripheral regions [8].

Research on the evaluation of the social performance of public sports facilities has undergone significant evolution. Equality is regarded as a fundamental value orientation, the highest moral standard, and a crucial value dimension pursued by modern society and political systems [9]. In the 1960s, Taitz’s public service facility location theory laid a solid theoretical foundation for studying the spatial layout of public service facilities and significantly advanced systematic research in this field. Based on Taitz’s theory, early research on the layout of public service facilities primarily focused on optimizing facility efficiency [10,11]. Over time, limitations in state-led public service provision have highlighted the growing role of market mechanisms as a driving force in the transformation of public service delivery. This shift has marked a transition from traditional state-led models to more flexible and efficient market-based supply models, triggering changes in research trends. For instance, Marxist geography, as represented by Harvey, emphasized the significance of capitalist production theory and urban location theory in the spatial service of public facilities [12]. Since the late 20th century, Western scholars have increasingly reflected on the shortcomings of previous welfare systems and advocated for the rights of citizenship and democratic participation. Rawls introduced the principle of fairness and equality of opportunity, arguing that society should ensure equal work opportunities and social status for individuals with the same abilities, skills, and motivations. From the 1970s to the 1990s, many developed Western countries adopted the “New Public Management” reforms to enhance the efficiency of social welfare systems [13]. The emergence of the “new public service” concept in urban planning has since elevated the humanistic value of public services to new heights, emphasizing citizen-centered service delivery. Evaluating the social performance of urban service distribution is now recognized as essential for achieving social equity and justice in public services. Social equity, as a concept, underscores the need to respect diverse demands and prioritize the interests of vulnerable groups. Broadly, the practice of evaluating social performance in urban services has evolved through three key stages: from “regional equality” to “social equity” and, ultimately, to “social justice” [14,15].

In recent years, advancements in geographic information systems (GIS), supported by big data technology, have been widely applied in smart city development, geographic information mapping, and spatial data collection and analysis [16]. These technologies provide robust support for theoretical and practical research related to public sports facilities [17,18].Based on this background, this study takes GIS as the basis, makes full use of multi-source geographic data, data integration is of great importance [19,20], aims to achieve fine spatial cognition of supply and demand of public sports facilities by constructing an accessibility model describing the relationship between spatial supply and public sports facilities demand. This study systematically analyzes the spatial distribution of Shanghai’s public sports facilities as well as accessibility at the dual scales of grids and streets from the perspective of supply and demand, and it analyzes the social performance of Shanghai’s public sports facilities in terms of regional equality and social fairness as well. The social performance of public sports facilities in Shanghai is measured in terms of regional equality and social equity. The findings of this study provide a scientific basis for optimizing and adjusting the spatial layout of public sports facilities in Shanghai. Furthermore, this research offers valuable insights for promoting the high-quality development of national fitness facilities in China.

## 2. Research data and research methodology

### 2.1. Data source and processing

**Fig 1:**
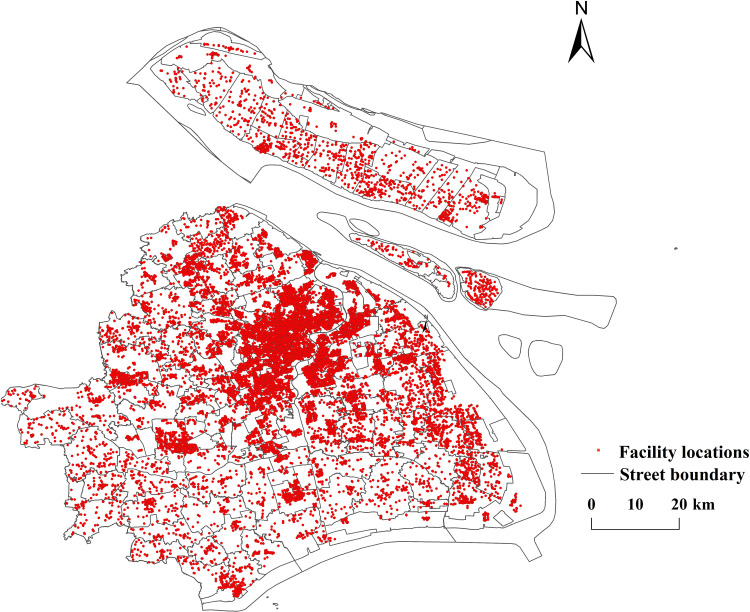
Distribution of public sports facilities in Shanghai (Reprinted from OpenStreetMap under a CC BY license, with permission from OpenStreetMap. https://www.openstreetmap.org/copyright/zh-TW).

The research data primarily consist of:

**(1) data on public sports facilities in Shanghai.** This study utilizes Python to crawl POI (Point of Interest) data from the Shanghai Public Sports Facilities Digital Management Service Platform. (http://www.shggty.com.cn/facilityMap.html) During the data collection process, strict data quality control principles were adhered to, cleaning data and identifing duplicates, missing values, and anomalies. Additionally, data consistency was ensured by writing scripts to verify the accuracy and completeness of the dataset. Spatial matching was conducted to guarantee that the data correspond to their actual geographic locations. Ultimately, data on public sports facilities in Shanghai was obtained. The data originates from the official statistics platform of the Shanghai Municipal Sports Bureau, and all public sports facilities in Shanghai are counted until 2023, making it highly representative of the current status of public sports facilities and authoritative. According to the classification standard set by the Shanghai Public Sports Facilities Digital Management Service Platform, the facilities are categorized into seven types, including 15,730 citizens’ puzzle and fitness courts, 1,166 citizens’ stadiums, 118 citizens’ gymnasiums, 1,241 citizens’ fitness trails, 43 citizens’ fitness centers, 46 citizens’ fitness stations, and 25 parents’ sports and health homes, totaling 18,369 facilities across all 16 administrative districts in Shanghai. the spatial distribution map of public sports facilities in Shanghai (see Fig 1) has been generated by the facility coordinate data imported into ArcGIS10.8.1, the tabular data converted into spatial vector data and the coordinate system projected as WGS_1984_UTM_Zone_48N.

**(2) Shanghai basic geographic information data.** The basic geographic information data for Shanghai primarily derives from OpenStreetMap (OSM). This includes data on the road network, county-level administrative divisions, and township (street) administrative divisions. The data is processed through steps such as extraction, correction, spatial matching, and other refinements to ensure accuracy and usability. These processed datasets are subsequently utilized for network analysis to support further research and applications.

**Fig 2:**
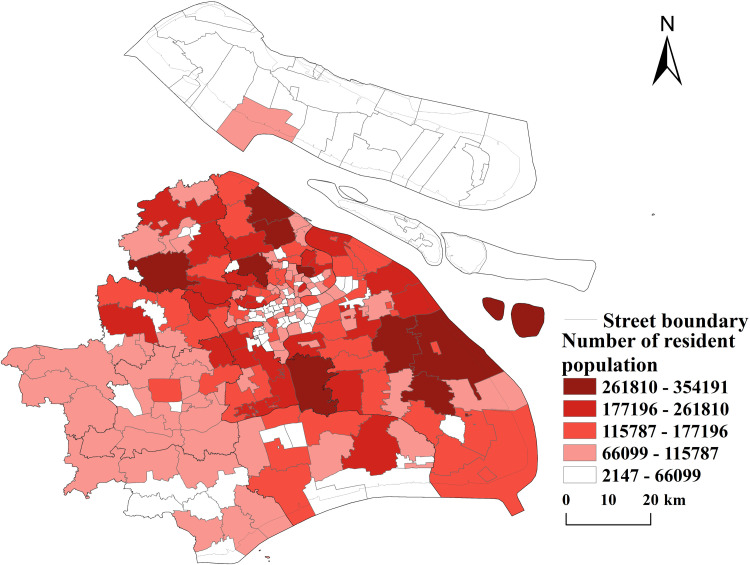
Spatial distribution of street-scale resident population size in Shanghai (Reprinted from OpenStreetMap under a CC BY license, with permission from OpenStreetMap. https://www.openstreetmap.org/copyright/zh-TW **).**

**Fig 3:**
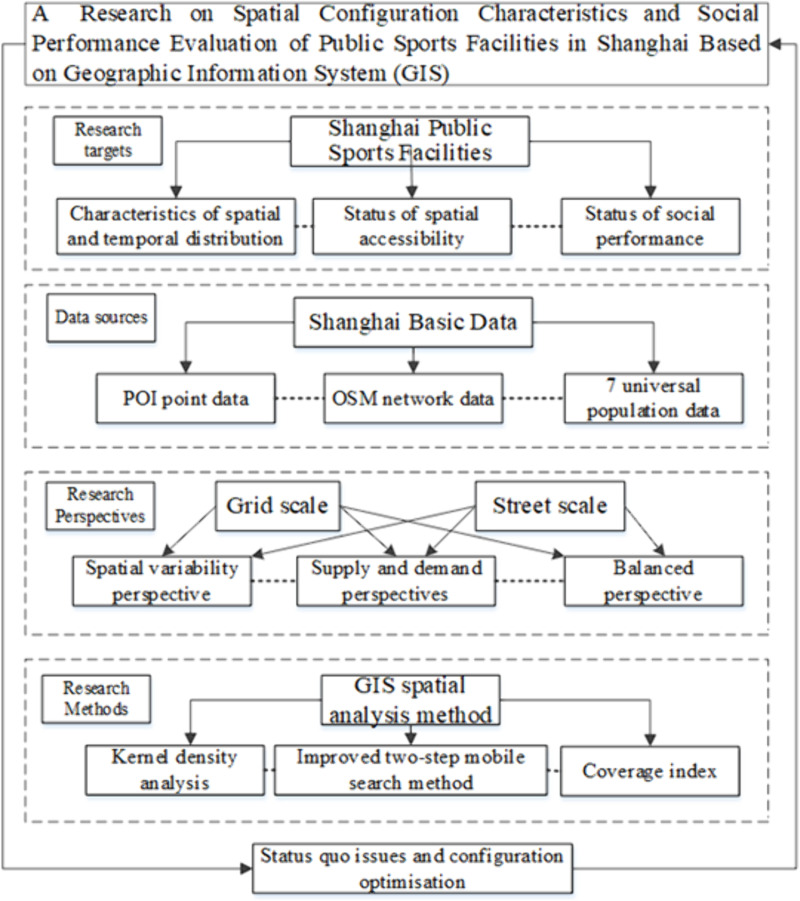
Technological route.

**(3) Population data of Shanghai.** The population density data for Shanghai, with a resolution of 1 km × 1 km, provided by WorldPop, serves as the primary basis for quantifying the supply and demand of public sports facilities in the city. Additionally, reference data includes the resident population data at the street scale in Shanghai, whose spatial distribution is derived from the bulletin of the seventh national population census of Shanghai (see Fig 2). The overall design framework and methodology adopted in this study are illustrated in Fig 3.

### 2.2. Research methods

This study employs several analytical methods to examine the spatial configuration of public sports facilities in Shanghai, including kernel density analysis, standard deviation ellipses, the Local Moran Index, and the Gaussian two-step moving search method. Additionally, social performance is evaluated by using the Gini coefficient, service radius coverage index, per capita service location entropy index, and questionnaire analysis. Among these methods, kernel density analysis method provides an initial overview of the spatial distribution of facilities, highlighting the spatial gaps in facility distribution. The standard deviation ellipse analysis further describes the directional and concentration trend of their distribution, offering insights into the overall spatial structure.The Local Moran Index building on the results from kernel density and standard deviation ellipse analyses, identifies specific hotspots and coldspots, revealing local spatial clustering and dispersion phenomena. The Gaussian two-step moving search method assesses the service level of facilities by calculating their spatial accessibility to residents in each region, offering foundational data for matching supply and demand in subsequent equity analysis. Finally, the Gini coefficient, service radius coverage index, and per capita service location entropy index are used to characterize the spatial allocation, and the fairness of the overall distribution is quantified. Based on the questionnaire analysis, the spatial distribution of public sports facilities across different regions is evaluated for balance, enabling an assessment of their social performance. These methods are complementary, forming a comprehensive analytical framework that systematically reveal the spatial allocation characteristics of public sports facilities in Shanghai, while also measuring their social performance from both a macro and micro perspective, considering distribution characteristics, accessibility and fairness.

#### 2.2.1. Spatial analysis methods.

**(1) Kernel density analysis method:** The kernel density analysis method is used to calculate the density of point elements around raster elements, based on the principle of weighting the influence of surrounding spatial points, it accurately depicts the distribution of facilities. The core mechanism involves considering each public sports facility as a central point and using a specific kernel function to determine its influence on the surrounding area. As the distance from the facility increases, this influence gradually diminishes. By superimposing the influence of all facilities, a continuous density surface is generated, enabling an accurate representation of the spatial distribution of facilities. The kernel density analysis offers several advantages over other spatial analysis methods, such as Tyson polygons: ① Weak model dependence: The method does not rely on specific data distribution assumptions. It can directly handle the original data of public sports facilities which may exhibit complex spatial distributions. Whether the data is uniformly distributed, discretely distributed, or characterized by significant spatial heterogeneity, kernel density analysis can adapt and yield accurate results. ② Strong robustness: When applied to the data of public sports facilities in Shanghai, kernel density analysis can still accurately capture the spatial distribution, even in the presence of various complex factors in the urban environment, as well as noise and outliers in the data. ③ Good sample fitting ability: By adjusting the bandwidth parameter, kernel density analysis can effectively fit spatial features at different scales. Smaller bandwidths emphasize local details, capturing clustering patterns in smaller areas, while larger bandwidths reveal overall spatial trends and facility distribution patterns on a larger scale. ④ Spatial density continuity: Unlike Tyson polygons, which divide the area around each facility point, creating clear boundaries between adjacent regions and resulting in discontinuous density changes, kernel density analysis produces a continuous spatial density surface by smoothing the data with the kernel function. This approach accurately reflects the transition from dense to sparse regions of facilities [21–26]. Based on these advantages, this study applies kernel density analysis to investigate the spatial distribution of public sports facilities in Shanghai.

The process of assessing the spatial clustering characteristics of public sports facilities in Shanghai consists of four steps: ① Determine the kernel function. The distribution of sports facilities data is relatively smooth and has no obvious local mutation or dispersion. The Gaussian kernel function has an infinite support domain, and it has non-zero weight for all distances, and the weight decreases rapidly with the distance, which can well simulate the spatial transition of the facilities in the distribution of the state, so this paper chooses the Gaussian kernel function. ② Determine the bandwidth. The points in the region are given different density weights, based on the concept of “ten-minute fitness circle” as the basic [27], according to the scope of influence of the public sports facilities in Shanghai and its spatial distribution to choose the bandwidth, which is 1 km. ③ Kernel density calculation.

The calculation formula is:


$$f(x,y)=\frac{1}{nh^{2}}\sum_{i=1}^{n}\;k(\frac{x-x_{i}}{h}\backslash$$ (1)

where f(x,y)represents the estimated kernel density at point x,y; n is the total number of public sports facilities in Shanghai; h is the bandwidth,a smoothing parameter that plays a critical role in balancing between overfitting and underfitting; k is the kernel function; and $\left(x_{i},y_{i}\right)$is the coordinate position of the facility. Firstly, the distance from each raster cell to the corresponding sports facility in the study area is calculated. Next, the density contribution of each facility point to the raster cell is computed using the kernel function $k\left(\frac{(x-x_{i}{)}^{2}+(y-y_{i}{)}^{2}}{h}\right)$ Finally, the kernel density value for the raster cell is obtained by summing up all the density contributions and multiplying the result by $\frac{1}{nh^{2}}$,. ④ The calculated kernel density values are assigned to their respective raster cells, and the results are visualized using ArcGIS 10.8.1 software.

**(2) Standard deviation ellipse analysis method:** In two-dimensional space, the distribution of public sports facilities in Shanghai exhibits two types of directional characteristics: isotropic, where the distribution is relatively uniform in all directions, and anisotropic, where the distribution varies significantly across directions [28]. Standard deviation ellipse analysis is employed in spatial analysis to evaluate the directional characteristics and degree of dispersion of public sports facilities. This method concentrates the distribution of facilities within a defined range, reflecting the dominant direction of their spatial and temporal patterns, and presents the results in the form of a standard deviation ellipse [29–31]. Compared to other methods, such as fractal dimension analysis, the standard deviation ellipse analysis method has notable advantages, including: ① Intuitivity: It provides a clear visualization of the distribution direction of Shanghai public sports facilities. By calculating the direction of the ellipse’s long axis, the overall trend in the dataset becomes evident and easy to interpret ② Spatial analysis ability: the size and center position of the ellipse effectively reflect both the concentration trend and the degree of dispersion of public sports facilities in Shanghai. The size of the ellipse indicates the degree of dispersion of the facilities, and the center position represents the average center of the facilities. ③ Data processing and application: This method simplifies the representation of geographic elements, facilitating rapid analysis and comparison of spatial characteristics across different datasets. Additionally, it evaluates the rationality of facility distribution based on the relative position of the ellipse and urban functional areas. Based on this, in order to characterize the directional distribution of public sports facilities in Shanghai, standard deviation ellipse analysis is introduced here.

The process of standard deviation ellipse analysis can be divided into five steps. ① Calculate the mean value of coordinates: calculate the mean value of x-coordinates $\overset{―}{X}=\frac{1}{n}\sum_{i=1}^{n}x_{i}$, by summing all x-coordinate values of public sports facilities in Shanghai whose sum will be divided by the total number of facilities n. The obtained $\overset{―}{X}$ is the average position of these facilities in the x-direction. Similarly calculate the average value of y-coordinate $\overset{―}{Y}=\frac{1}{n}\sum_{i=1}^{n}y_{i}$, adding up the y-coordinate values of all facilities whose sum will be divided by n. The result represents the average position of the facilities along the the y-axis. This ($\overset{―}{X}\overset{―}{,Y})$point is the center position of the standard deviation ellipse, which roughly reflects the central distribution area of public sports facilities in Shanghai in the plane. ② Calculate the standard deviation.

The formula is:


$$\begin{array}{cc} SDE_{x} & =\sqrt{\frac{\sum_{i=1}^{n}\;(x_{i}-X―{)}^{2}}{n}}\\ SDE_{y} & =\sqrt{\frac{\sum_{i=1}^{n}\;(y_{i}-Y―{)}^{2}}{n}} \end{array}$$ (2)

For each public sports facility in Shanghai, the square of the difference between its x-coordinate and the meanx-coordinate $\overset{―}{X}$ $(x_{i}-X―{)}^{2}$ is computed, The squared differences are summed, divided by the total number of facilities n, and the square root is then calculated. The $SDE_{x}$value represents the degree of dispersion of public sports facilities in the x direction. Similarly, the square differences between each facility’s y-coordinate and the mean y-coordinate $\overset{―}{Y}$ $(y_{i}-Y―{)}^{2}$ are summed, divided by n, and the square root is taken. $SDE_{y}$ reflects the degree of discretization of the facilities in they-direction. ③Calculate the covariance. $S_{xy}=\frac{1}{n}\sum_{i=1}^{n}\;\left(x_{i}-X―\mathrm{\backslash rightleft}(y_{i}-Y―\right)$. The covariance values quantify the interrelationship between the x and y directions of public sports facilities in Shanghai. ④ Calculate the long axis, short axis and rotation angle of the ellipse. Let $\lambda_{1}$ and $\lambda_{2}$ be the eigenvalues of the matrix $\left[\begin{array}{cc} SDE_{x}^{2} & S_{xy}\\ S_{xy} & SDE_{y}^{2} \end{array}\right]$ with $\lambda_{1}⩾\lambda_{2}$. The lengths of the long($a=\sqrt{\lambda_{1}})$,and short ($b=\sqrt{\lambda_{2})}$axes represent the primary and secondary distribution ranges of public sports facilities in different directions. The direction of the long axis corresponds to the most dispersed distribution of facilities, while the short axis indicates the most concentrated direction. Calculate the rotation angle θ of the ellipse. Let $\vec{v}$ be the eigenvector corresponding to $\lambda_{1}$ and $\theta =\frac{1}{2}\arctan\left(\frac{2S_{xy}}{SDE_{x}^{2}-SDE_{y}^{2}}\right)$. The rotation angle θ represents the clockwise rotation angle of the long axis relative to true north, indicating the orientation of the primary distribution direction of public sports facilities in Shanghai.⑤Visualization of results. The center of the ellipse ($\overset{―}{X}\overset{―}{,Y})$, the long axis a, the short axisb and the rotation angle θ are input into ArcGIS 10.8.1 to visualize the standard deviation ellipse and analyze the directional distribution characteristics of public sports facilities in Shanghai.

**(3) Spatial autocorrelation analysis:** Spatial autocorrelation analysis examines the degree of correlation between spatial samples and their surrounding samples, reflecting their distribution characteristics. It is primarily divided into global autocorrelation and local autocorrelation [32–37]. Local Moran’s I effectively captures spatial heterogeneity, which refers to the differences in attribute values across spatial units and their spatial distribution. This helps to gain a deeper understanding of the complex structure and distribution characteristics of spatial data [38]. It classifies the study area into four categories: High-High Clusters, Low-Low Clusters, High-Low Outliers, and Low-High Outliers.Low-Low Clusters, High-Low Outliers, and Low-High Outliers. Compared with Ripley’s K-function and other analytical methods, the local Moran index has the following three advantages. Precise localization: By applying the Local Moran Index, the spatial correlation pattern between the attribute values of public sports facilities in Shanghai and their neighboring units can be thoroughly explored. This enables accurate identification of spatial hotspots (High-High Outliers), coldspots (Low-Low Outliers), and High-Low and Low-High outlier areas. ② Flexibility: Different spatial weight matrices, such as neighbor weights and distance weights, can be chosen based on research objectives and data characteristics, which is able to adapt to various types of spatial analysis.③Intuitive visualization: The Local Moran Index intuitively displays local autocorrelation across spatial units by generating LISA cluster maps.In the map, different colors or symbols represent various clustering patterns, providing an intuitive understanding of spatial distribution characteristics.Therefore, in order to characterize the spatial heterogeneity of public sports facilities in Shanghai, the local Moran index is introduced here.

The process of analyzing the local Moran index is divided into three steps. ① Calculate the mean value of attributes of each spatial unit of Shanghai public sports facilities. Firstly, a spatial weight matrix is constructed based on neighboring relationships. Then, the mean attribute value of public sports facilities within each spatial unit is calculated, denoted by x―. The mean attribute value x― is calculated as $x―=\frac{\sum_{i=1}^{n}\;x_{i}}{n}$, where$x_{i}$represents the attribute value of public sports facilities in the i c -th spatial unit, and n is the total number of spatial units ② Perform specific calculations:

The calculation formula is:


$$I_{i}=\frac{\left(x_{i}-x―\right)}{S^{2}}\sum_{j=1}^{n}\;w_{ij}\left(x_{j}-x―\right)$$ (3)

WhereI is the Moran index$,x_{i}$ is the attribute value of public sports facilities of spatial unit i; x― is the average value of public sports facilities attributes of all spatial units calculated earlier; $S^{2}=\frac{1}{n}\sum_{i=1}^{n}\;(x_{i}-x―{)}^{2}$ measures the variance of the attribute values, reflecting the degree of data dispersion.; and $w_{ij}$ represents the weight between spatial units i and j in the weight matrix constructed earlier.. weights between spatial cells i and j in the weight matrix constructed earlier; n is the total number of spatial cells. ③ Interpretation of results: when $I_{i}$＞0, if $x_{i}$＞x―, the attribute value of public sports facilities in this spatial unit is above average, and the surrounding units also have higher values, indicating a High-High cluster (hotspot), which indicates that the sports facilities in this community and the surrounding area are better in terms of quantity and quality, and it is an advantageous area for the distribution of public sports facilities. If $x_{i}$＜x―, the attributes of public sports facilities in this spatial unit are below average, and the neighboring units also have lower values, forming a Low-Low cluster (cold spot), which indicates that there is a relative lack of sports facilities in this area and its surrounding areas, and that this is an area that needs to be focused on and improved. When $I_{i}$＜0, if $x_{i}$＞x―, it indicates that the value of the attribute of public sports facilities in this spatial unit is higher than the average level, but the value of the surrounding units is smaller, and there is a high - low clustering, which reflects that although the sports facilities in this region are relatively good, the surrounding supporting facilities are insufficient, and it can be considered to strengthen the radiation-driven role of the facilities. If $x_{i}$＜x―, i.e., the value of the attributes of sports facilities in this spatial unit is lower than the average level, while the value of the surrounding units is larger, presenting a low-high clustering, which suggests that the region can be allowed to make better use of the resources of the surrounding sports facilities through inter-regional cooperation and sharing and other means. When $I_{i}$ = 0, there is no significant spatial autocorrelation between this spatial unit and its neighbors, suggesting a random distribution, and the layout can be optimized by comprehensively considering its connection with different types of surrounding areas in planning and other aspects.

**(4) Spatial accessibility analysis:** Accessibility research originates from classical location theory, which aims to evaluate the locational advantages or disadvantages of spatial elements, such as points, lines, or regions [39], and the main research methods include buffer analysis, two-step moving search method, etc., among which the two-step floating catchment area method (2SFCA) is essentially a specific application of the gravity model, and its basic principle is to search and calculate the spatial accessibility of infrastructure in two steps [40–42]. The Gaussian two-step moving search method improves upon the traditional two-step moving search method by introducing a Gaussian distance decay function, offering the following advantages. ① Accurate portrayal of the distance decay effect: in a mega city like Shanghai, the distance relationships between different areas and public service facilities are complex and diverse. By incorporating the Gaussian distance decay function, this method accurately simulates how the accessibility of public sports facilities diminishes as distance increases. Unlike methods that overlook or simplify the distance decay effect, this approach avoids using a fixed radius (e.g., buffer analysis) and instead measures accessibility dynamically based on actual distances to infrastructure. ② Comprehensive consideration of supply and demand: this method comprehensively considers both supply-side and demand-side factors in calculating accessibility. On the supply side, it accounts for factors such as the location, size, and service capacity of infrastructure; on the demand side, it considers key elements like population distribution.Compared to methods that focus exclusively on either the supply side (e.g., buffer analysis based on facility locations) or the demand side (e.g., approaches emphasizing population mobility paths), the Gaussian two-step moving search method provides a more realistic representation of spatial accessibility. ③ Effective adaptation to spatial heterogeneity: Shanghai’s urban space is highly heterogeneous, including different topography, land use types, transportation networks, and population densities. The Gaussian two-step moving search method effectively adapts to spatial heterogeneity. For example, in old urban areas such as Huangpu District, the population is dense and traffic congested, but the public sports facilities may be relatively few and unevenly distributed; whereas in new urban areas such as Minhang District, the population is relatively sparse in some areas, although the area is large. This method evaluates the accessibility of public sports facilities based on regional characteristics, providing robust support for differentiated urban planning and resource allocation strategies [43–50]. Based on this paper, Gaussian two-step moving search method is adopted to evaluate the accessibility of public sports facilities in Shanghai from grid scale and administrative district scale. The specific calculation steps are as follows:

Step 1: Supply Evaluation (Centred on Sports Facility Points)
  ① Defining service search range: For each sports facility pointj, define a distance-based service search range centred on the time threshold $d_{0}$.
  ②Gaussian weight assignment: For each residential neighbourhood point k, assign weights using the Gaussian equation. The Gaussian weight $g\left(d_{kj},d_{0}\right)$ is calculated as:

$$g\left(d_{ki},d_{0}\right)=e^{-\frac{d_{kj}^{2}}{2d_{0}^{2}}}$$ (4)

③Calculate the weighted sum of demand sizes: For each sports facility point j, calculate the weighted sum of demand sizes within its service area by multiplying the population of each cell by its corresponding Gaussian weight and summing up the results.
④Calculate the supply-demand ratio $R_{j}$: For each sports facility point j, calculate $R_{j}$, by dividing its service size by the weighted demand size.

The formula is:


$$R_{j}=\frac{s_{j}}{\Sigma_{k\in \left\{d_{kj}\leq d_{0}\}\right\}}g\left(d_{kj},d_{0}\right)D_{k}}$$ (5)

Here $s_{j}$ represents the size of the sports facility point, $D_{k}$ is the population of the residential community point k within the search range, $d_{kj}$ denotes the travel time between the demand point k and the service provision point j, and $g\left(d_{kj},d_{0}\right)$ is the Gaussian time decay weight for the population of the residential community point k, and the Gaussian time decay function is expressed as:


$$R_{j}=\frac{s_{j}}{\Sigma_{k\in \left\{d_{kj}\leq d_{0}\}\right\}}g\left(d_{kj},d_{0}\right)D_{k}}$$ (6)

Step 2: Demand evaluation (centred on residential plot points)
①Define spatial demand range: For each residential community point k, define a spatial demand range centred on $d_{0}$ as the time threshold.
②Gaussian weight assignment: For each sports facility point j, assign weights by using the Gaussian equation. The Gaussian weight $g\left(d_{kj},d_{0}\right)$ can be calculated by the following equation:

$$g\left(d_{ki},d_{0}\right)=e^{-\frac{d_{kj}^{2}}{2d_{0}^{2}}}$$ (7)

③Calculate accessibility $A_{k}$: For each residential neighbourhood point k, calculate the weighted sum of the supply-demand ratios of all sports facility points within the spatial demand range of $d_{0}$. Multiply the supply-demand ratios of each sports facility point by its corresponding Gaussian weight, then sum the results.The formula is expressed as:

$$A_{k}=\sum i\in \{d_{kj}\leq d_{0}\}\}\;g\left(d_{ij,}d_{0}\right)R_{j}$$ (8)

where $\sum i\in \{d_{kj}\leq d_{0}\}\;$ denotes the summation of all i satisfying $d_{kj}\leq d_{0}$ at spatial demand range with $d_{0}$ as the time threshold; $g\left(d_{ij,}d_{0}\right)$ is the Gaussian weight function where $d_{ij,}$,is the residential neighbourhood point k to the travelling time to the sports facility pointj, $d_{0}$ is the time threshold; and $R_{j}$ is the ratio of supply and demand of the sports facility point j.

Using the Gaussian two-step moving search method, the accessibility of public sports facilities in Shanghai is evaluated by integrating POI data of sports facilities with basic geographic information. The supply of public sports facilities is represented by spatial data such as the density or number of facilities within 1km grid cells. Then, based on the population data and basic geographic information data of Shanghai, the spatial distribution density of the population in the 1km grid cells is calculated and used as the demand for public sports facilities. Finally, the supply and demand as well as the service radius are inputted into the model of Gaussian two-step moving search method, so as to realize the accessibility evaluation of public sports facilities in Shanghai.

#### 2.2.2. Social performance assessment methods.

The Gini coefficient, known for reflecting social equity, has been applied by scholars to assess the distribution of public resources and the layout of public facilities. [51]. For example, a Gini coefficient <0.2 represents a high degree of equality; 0.2–0.29 is relatively equal; 0.3–0.39 is moderately reasonable; 0.4–0.59 represents a significant disparity; and ≥ 0.6 indicates an extremely wide gap [52]. Next, the number of public sports facilities per capita was ranked in descending order according to the number of public sports facilities per spatial unit in the study area. The total resident population was then divided into intervals, each representing 10% of the total resident population. By calculating the proportion of public sports facilities utilized within each population interval and presenting it graphically using the Lorenz curve, the distribution pattern of public sports facilities across the entire resident population can be visualized. The Lorenz curve not only enhances the interpretation of the Gini coefficient but also provides deeper insights into the inequality in access to public sports facilities among residents. While the Gini coefficient and Lorenz curve are effective in characterizing social equity, they face limitations in describing the spatial distribution of public facilities.For this reason, this paper takes the Gini coefficient and Lorenz curve as the basis, refers to the social performance evaluation methods of urban public services such as green space and transportation [52,53], and establishes the social performance evaluation system of public sports facilities, with “regional equity” and “social equity” as the two key dimensions. In order to measure the distribution of these two dimensions, the two core indicators of “service radius coverage” and “per capita service location entropy” are used. The “service radius coverage” and “per capita service location entropy” indicators capture micro-level information, such as the convenience of facility access for individual residents. In contrast, the Gini coefficient and Lorenz curve offer macro-level insights into the fairness of resource distribution across regions. The integration of these indicators facilitates a more comprehensive assessment of the social performance of public sports facilities.For instance, the Gini coefficient might indicate equitable distribution of sports facilities between two regions. However, the service radius coverage may reveal that geographic barriers (e.g., mountain ranges, rivers) limit physical accessibility in one region. A comprehensive framework for measuring social performance is attained by combining the Lorenz curve and the Gini coefficient.

(1) Lorenz Curve and Gini Coefficient: The advantage of simplicity of the Lorenz Curve and the Gini Coefficient is clearly demonstrated in the international metropolis of Shanghai. The combination of the Lorenz curve and the Gini coefficient can provide a comprehensive view of social performance measurement. The visual display of the Lorenz curve quickly grasps the general trend, while the quantitative value of the Gini coefficient can accurately measure the extent of this trend. The specific analysis process is as follows. ①Constructing the Lorenz Curve: First, rank each administrative district by the number of public sports facilities per capita (total facilities divided by resident population) from low to high, then calculate the cumulative population share and cumulative number of facilities share of each administrative district, and finally draw the Lorenz Curve with the cumulative population share as the horizontal axis and the cumulative number of facilities share as the vertical axis. ②Calculate the Gini coefficient: Calculate the area between the Lorenz curve and the line of absolute equality (diagonal) A, and the area to the right of the lower part of the Lorenz curve, B. Substitute the formula: $G=\frac{A}{A+B}$.
(2) Service Radius Coverage Indicator: Shanghai, as an economically developed and populous city, exhibits significant disparities in the distribution of public sports facilities across its regions.The service radius coverage indicator can visualize the spatial service scope of each sports facility and the degree of coverage of the surrounding residents. The analysis process is as follows:
the calculation formula is:

$$R_{i}=\frac{\sum_{j=1}^{n}\;s_{j}-S_{a}}{s_{i}}$$ (9)

①Calculate the total area covered by each facility. According to the formula $\sum_{j=1}^{n}\;s_{j}$, the area of the community covered by the service radius of all public sports facilities in the community of unit i is summed up. Then subtract the overlapping community area $S_{a}$ covered by the service radius of community sports and fitness venues from the above sum.
②Calculate the service radius coverage rate: divide the result of the numerator part by the area of the community in unit i, $s_{i}$, to obtain the service radius coverage rate of community sports and fitness venues in unit i, $R_{i}$.

These steps calculate the service radius coverage rate of public sports facilities in each unit, enabling an assessment of spatial distribution parity. (3) The per capita service location entropy indicator evaluates the balance between public sports facilities and population distribution in Shanghai, assessing whether services are equitably distributed across densely populated areas. The specific analysis process is as follows:

The formula is:


$$LD_{i}=\frac{\frac{Z_{in}}{P_{i}}}{\frac{Z_{an}}{P_{a}}}$$ (10)

①Calculate the number of facilities per capita in each region: according to the formula$\frac{Z_{in}}{P_{i}}$, divide the number of public sports facilities $Z_{in}$ in each administrative region of Shanghai by the number of population $P_{i}$ in each administrative region.
②Calculate the number of sports facilities per capita in Shanghai: according to the formula $\frac{Z_{an}}{P_{a}}$, divide the total number of public sports facilities in Shanghai, $Z_{in}$, by the total population of Shanghai, $P_{a}$.
③Calculate the location entropy of each region: According to the formula, substitute the number of sports facilities per capita in each region (numerator part) and the number of sports facilities per capita in Shanghai (denominator part) into the formula to calculate the location entropy of each region.

Through the above steps, the location entropy of per capita services in each region of Shanghai can be calculated, which in turn compares the relative saturation degree of public sports facilities in different regions and evaluates the equilibrium of public sports facilities in spatial distribution

## 3. Results and analyses

### 3.1. Spatial features analyses

**Fig 4:**
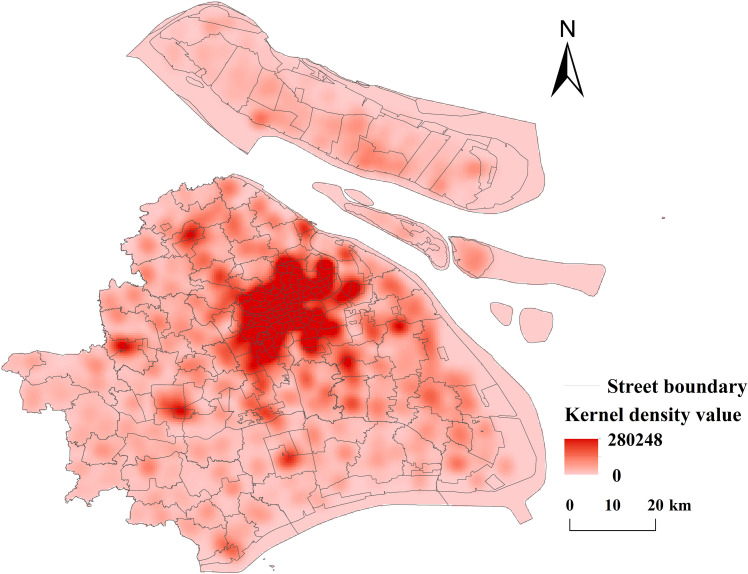
Distribution of nuclear density of public sports facilities in Shanghai (Reprinted from OpenStreetMap under a CC BY license, with permission from OpenStreetMap. https://www.openstreetmap.org/copyright/zh-TW).

**(1) Kernel Density Analysis:** Kernel density analysis and spatial visualisation of public sports facilities in Shanghai were carried out with GIS software (see Fig 4).

**Fig 5:**
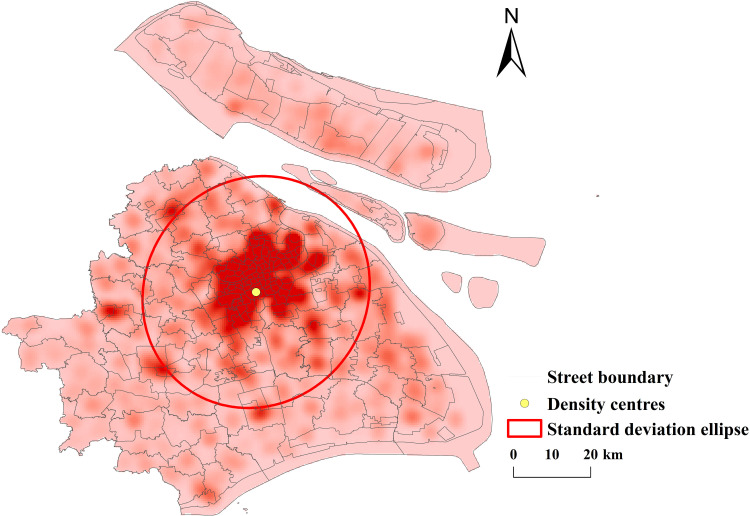
Distribution center and development direction of public sports facilities in Shanghai (Reprinted from OpenStreetMap under a CC BY license, with permission from OpenStreetMap. https://www.openstreetmap.org/copyright/zh-TW).

①Spatial Distribution Structure: The spatial distribution of public sports facilities in Shanghai exhibits a distinct “central clustering + peripheral clustering” pattern. In downtown Shanghai, districts such as Jing’an (1,472 facilities), Hongkou (467 facilities), Yangpu (665 facilities), Huangpu (406 facilities), Xuhui (646 facilities), Changning (429 facilities), and Putuo (825 facilities) form a multi-center cluster. In contrast, peripheral districts such as Jiading, Qingpu, Songjiang, and Pudong New District show a scattered distribution trend with localized concentrations and a broader radiation range. ② Coupling Analysis: By overlaying the scatter map of public sports facilities in Shanghai with the city’s population density map, it is evident that the spatial distribution of public sports facilities generally aligns with the population distribution trends. Central Urban Areas: The central city exhibits higher population densities, with a corresponding concentration of public sports facilities. Fengxian District: The population peaks in the northwest, while the eastern region has lower population densities. The distribution of public sports facilities closely corresponds to these patterns. Songjiang District: The population is primarily concentrated in the center, and the distribution of public sports facilities is well-aligned with this pattern. Baoshan District: The population density peaks in the northeast, but the distribution of public sports facilities is more centralized, showing a slight deviation. Jinshan District: The population is mainly distributed in the northwest and southeast, with a relatively dispersed, point-like pattern. The distribution of public sports facilities aligns broadly with this spatial trend. These analyses highlight a strong spatial coupling between public sports facilities and population distribution in Shanghai, characterized by the distinct spatial distribution pattern of “central clustering + multi-point agglomeration.” (2) Standard deviation ellipse analysis:

The spatial distribution of public sports facilities in Shanghai was analyzed using the standard deviation ellipse method in GIS software to examine the degree of dispersion and directionality in the spatial plane. The results are as follows: Long axis: 30,085.4211,Short axis: 25,029.0322,Flatness (difference between long and short axes): 5,056.3889,Direction angle: 11.5915°,Mean center coordinates: (351783.181183, 3452572.91312).The spatial directionality of public sports facilities in Shanghai extends along the northeast-southwest axis, as indicated by the long axis of the ellipse. The spatial layout shows a trend of higher concentrations of public sports facilities in the north-south direction and fewer in the east-west direction. This reflects a distribution pattern radiating from the central urban area toward the north-south axis (see Fig 5).

**Fig 6:**
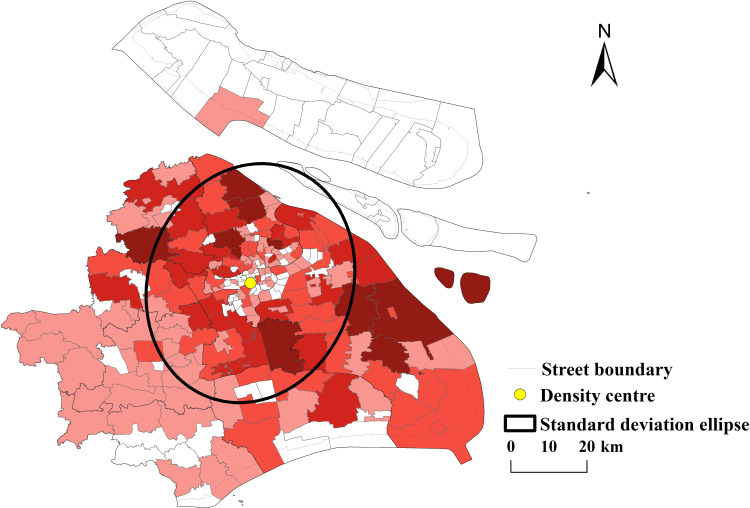
Distribution centers and development direction of Shanghai residents (Reprinted from OpenStreetMap under a CC BY license, with permission from OpenStreetMap. https://www.openstreetmap.org/copyright/zh-TW).

②Coupling Analysis of Public Sports Facilities and Population Distribution: The standard deviation ellipse analysis was also conducted for Shanghai’s population distribution to compare its spatial characteristics with those of public sports facilities. The results for the resident population are as follows: Long axis: 31,527.1199,Short axis: 23,206.4207,Flatness: 8,320.6992,Direction angle: 10.9897°,Mean center coordinates: (351506.167545, 3453692.32666),Compared with the standard deviation ellipse of public sports facilities, the population distribution in Shanghai exhibits a similar northeast-southwest directional trend, as the difference in the direction angles of the two ellipses is minimal. However, the population distribution has a greater flatness, indicating that it is more prominently distributed along the northeast-southwest axis. The shorter short axis of the population ellipse reflects a stronger centripetal force in the spatial distribution of residents (see Fig 6).

**Fig 7:**
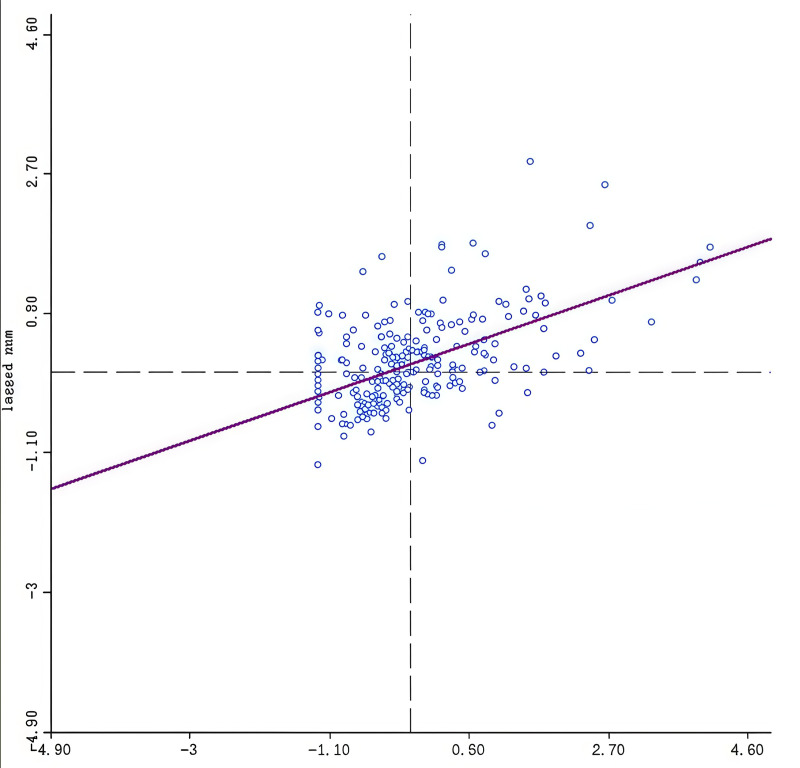
Scatterplot of Moran Index for Public Sports Facilities in Shanghai.

**Fig 8:**
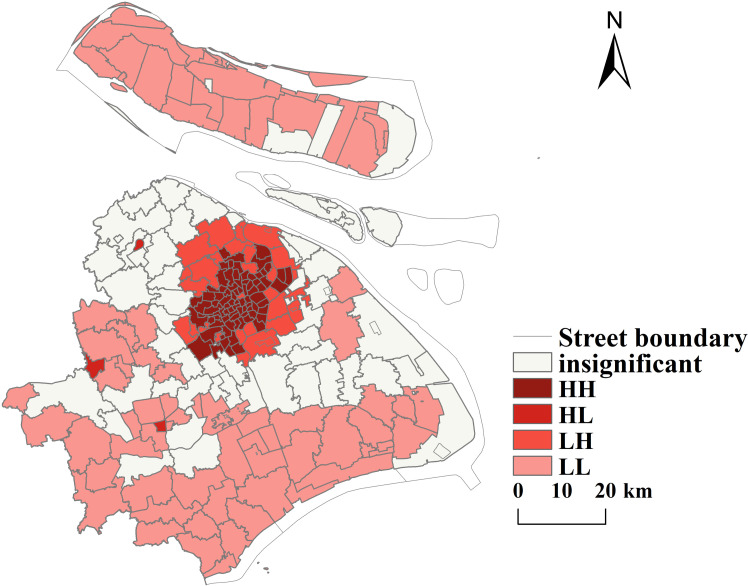
Shanghai Public Sports Facilities LISA Cluster Map (Reprinted from OpenStreetMap under a CC BY license, with permission from OpenStreetMap. https://www.openstreetmap.org/copyright/zh-TW).

The results of the standard deviation ellipse analyses indicate a relatively strong spatial coupling between public sports facilities and population distribution in Shanghai. Both distributions show clear northeast-southwest directional characteristics, with public sports facilities aligning well with the population density trends. This alignment suggests an effective spatial coupling between the supply of public sports facilities and the demand represented by population distribution. (3) To further explore the spatial distribution differences of public sports facilities in Shanghai, GeoDa 1.16 software was used to generate a Moran’s Index scatter plot and a LISA (Local Indicators of Spatial Association) cluster map. The analysis was conducted under the premise of a 5% significance level. The results revealed four key characteristics of the local spatial autocorrelation of public sports facilities in Shanghai:

①High-High Agglomeration (HH): High-high agglomeration areas are regions where the agglomeration level of public sports facilities is high, and the neighboring areas also exhibit high levels of agglomeration. These areas are primarily concentrated in the central city circle of Shanghai. Specific locations include: Jing’an District:Republican New Road Street, Yichuan Road Street, and West Tianmu Road Street. Putuo District: Ganquan Road Street, Changshou Road Street, and Changfeng Xincun Street.Changning District: Huayang Road Street and Tianshan Road Street.Xuhui District: Xujiahui Street and Fenglin Road Street. Huangpu District: Laosimen Street and The Bund Street. Hongkou District: Lujiazui Street and Jiaxing Road Street. These areas demonstrate a high concentration of public sports facilities, particularly within Shanghai’s central urban core (see Figs 7 and 8).② Low-Low Agglomeration (LL):Low-low agglomeration areas are regions where both the agglomeration level of public sports facilities and that of their neighboring areas are low. These areas are mainly distributed in Shanghai’s peripheral urban areas, including: Qingpu District: Jinze Town and Baihe Town. Jinshan District: Lvxiang Town and Huimin Street. Fengxian District: Zhuangxing Town and Qingcun Town. Pudong New District: Chuansha New Town and Wanxiang Town. Chongming District: Sanshing Town and Zhongxing Town. These regions exhibit small differences in the level of agglomeration, with consistently low levels across the identified areas.③ Low-High Agglomeration (LH):Low-high agglomeration areas represent regions where public sports facilities exhibit low levels of agglomeration, while neighboring areas show high levels. These areas are primarily located in: Baoshan District: Wusong Street and Gucun Town. Pudong New District: Yangjing Street and Shanggang Xincun Street. Minhang District: Xudiao Town and Qibao Town. These areas highlight significant disparities between the local and neighboring levels of facility agglomeration.④ High-Low Agglomeration (HL):High-low agglomeration areas are regions with high levels of public sports facility agglomeration, surrounded by neighboring areas with low levels of agglomeration. These areas are mainly concentrated in: Jiading District: Jiading Town Street. Songjiang District: Yueyang Street. The high concentration levels in these areas demonstrate a unipolar pattern, with minimal trickle-down effects on adjacent streets and towns.From a comprehensive perspective, the spatial distribution of public sports facilities in Shanghai shows clear characteristics of “central clustering + multi-point concentration”. The central urban areas exhibit a high level of concentration, while the peripheral urban areas have significantly lower levels of facility agglomeration, reflecting an uneven spatial distribution pattern.

**(4) Sensitivity analysis of spatial features:** To further verify the reliability and robustness of the spatial characterization results, a sensitivity analysis of the results from the three methods is conducted. The consistency and differences among the results of multiple methods are comprehensively analyzed to validate the spatial distribution characteristics of public sports facilities in Shanghai (see Table 1).

**Table 1:** Comparison table of spatial characterization of public sports facilities in Shanghai.

Method	Hot spot areas	Cold spot areas	Directionality	Characteristics
Kernel density analysis	Jing’an and Changning districts, etc. are core hotspot areas	Not highlighting cold spots	Not highlighting directionality	Demonstrating the overall trend, emphasizing the continuity of density distribution
Local Moran index	Jing’an and Huangpu districts, etc. are high - high aggregation area	Chongming and Jinshan districts, etc. are low - low aggregation areas	Not highlighting the directionality	Emphasizing the local prominence, cold hotspots can be identified
Standard deviation ellipse	Jing’an district, Huangpu district, etc are distribution centers	Not highlighting cold spots, with rounded direction	Distribution is concentrated in the central city,	Provides overall distribution direction and concentration trend

①Consistency analysis: First, the consistency of core hotspot areas: Jing’an District and Huangpu District are identified as core hotspot areas across all three methods. Second, the consistency in spatial distribution trends: the three methods collectively revealed that Shanghai’s public sports facilities exhibit a pattern of being ‘concentrated in the central urban areas and dispersed in peripheral regions. Finally, the analysis confirmed a strong coupling between the distribution of facilities and population distribution across the three methods.

Differential analysis: First, differences were observed in the hotspot ranges: the kernel density analysis exhibited a larger hotspot range, whereas the high-high clustering range identified by the local Moran index was smaller. Second, differences emerged in the identification of cold and hot spots: among the three methods, only the local Moran index placed greater emphasis on identifying cold spots. Finally, directional differences were noted: while kernel density analysis and the local Moran index primarily focused on the spatial distribution of hotspots and cold spots, the standard deviation ellipse clarified the directional characteristics of facility distribution by comparing the long and short axes.

In summary, the results of the spatial characterization of public sports facilities in Shanghai have strong robustness.

### 3.2. Spatial accessibility analysis

**Fig 9:**
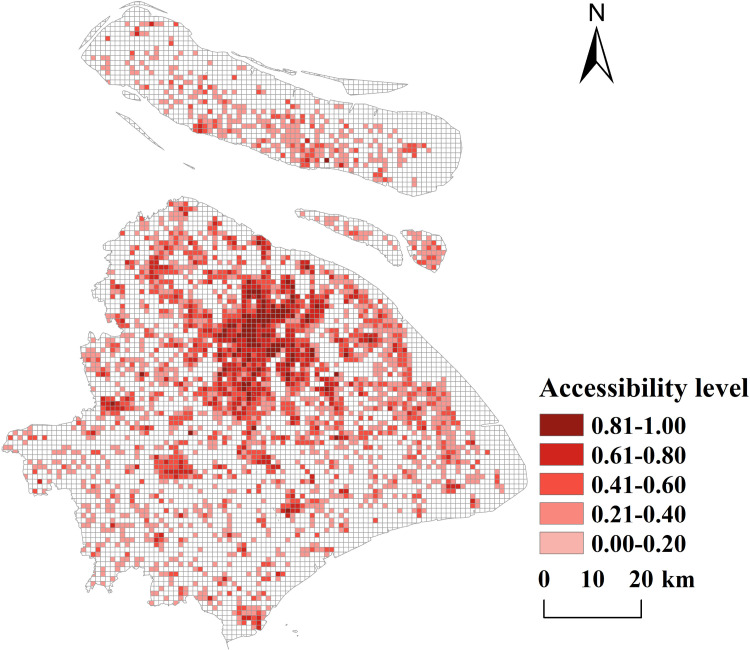
Accessibility map of public sports facilities in shanghai at grid scale (Reprinted from OpenStreetMap under a CC BY license, with permission from OpenStreetMap. https://www.openstreetmap.org/copyright/zh-TW).

**Fig 10:**
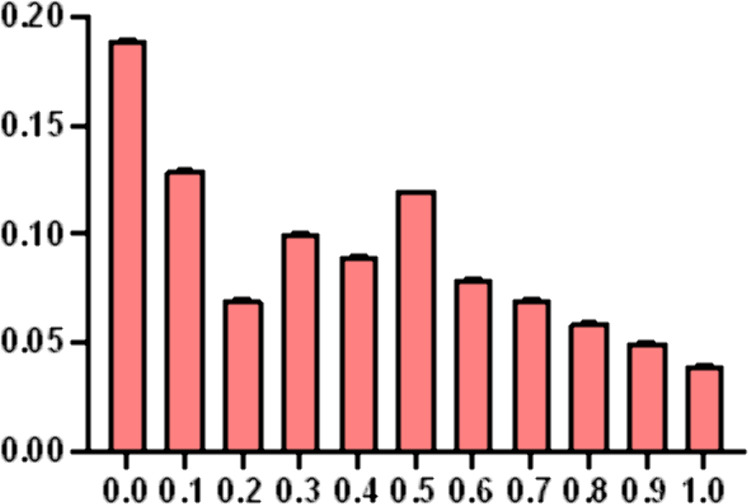
Statistical distribution of normalized accessibility of public sports facilities at grid scale in Shanghai Municipality.

**(1) Spatial accessibility analysis at grid scale:** Using the Gaussian two-step moving search method and GIS software, the spatial distribution of normalized accessibility for public sports facilities in Shanghai was calculated at a 1 km grid scale. The accessibility values were classified into five categories based on natural discontinuities (see Fig 9).From the frequency histogram of spatial accessibility (see Fig 10), the distribution is observed to be positively skewed overall. The largest frequency interval is within the range of [0, 0.05]. The left half of the frequency distribution exhibits significant fluctuations, while the right half is relatively stable, with a longer tail on the right compared to the left.In summary, the accessibility of public sports facilities in Shanghai demonstrates uneven spatial distribution characteristics, with notable disparities across different regions.

**Fig 11:**
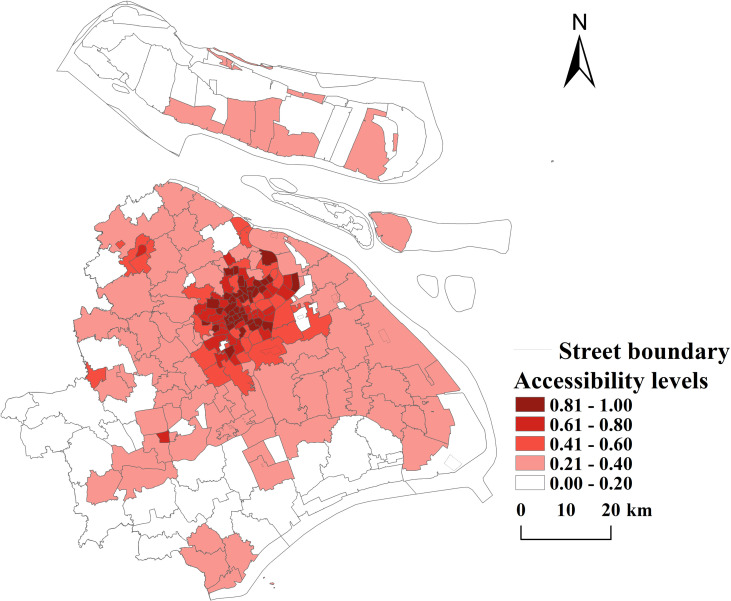
Results on the accessibility of public sports facilities at the scale of Shanghai’s administrative divisions (Reprinted from OpenStreetMap under a CC BY license, with permission from OpenStreetMap. https://www.openstreetmap.org/copyright/zh-TW).

**(2) Spatial accessibility analysis at street scale:** Building on the results of the grid-scale analysis, spatial accessibility was further averaged and aggregated to the street scale (see Fig 11). The streets with the highest accessibility in Shanghai are: Caojiadu Street (accessibility: 0.8775) in Jing’an District. Taipuqiao Street (accessibility: 0.6826) in Huangpu District. Ganquan Road Street (accessibility: 0.662) in Putuo District. Linfen Road Street (accessibility: 0.6572) in Jing’an District. Cao Yang Xincun Street (accessibility: 0.6523) in Putuo District. These high-accessibility streets are primarily concentrated in Jing’an District, demonstrating a strong correspondence between high-value agglomerations at the street and grid scales. Conversely, the streets with the lowest accessibility are: Waigaoqiao Free Trade Zone (accessibility: 0.00087) in Pudong New Area. Jinqiao Township (accessibility: 0.0011) in Pudong New Area. Changxing Township (Qianwei Farm) (accessibility: 0.0045) in Chongming District. Dongping Township (accessibility: 0.0073) in Chongming District. Nanhui New Township (accessibility: 0.0075) in Pudong New Area. The low-accessibility streets are predominantly located in Pudong New Area, with additional examples in Chongming District.

A statistical analysis of accessibility at the district level reveals significant spatial disparities. The districts, ranked by their average accessibility values in descending order, are as follows: Jing’an District ＞ Hongkou District ＞ Huangpu District ＞ Putuo District ＞ Changning District ＞ Xuhui District ＞ Yangpu District ＞ Baoshan District ＞ Pudong New Area ＞ Minhang District ＞ Jiading District ＞ Songjiang District ＞ Qingpu District ＞ Fengxian District ＞ Chongming District ＞ Jinshan District (as shown in Table 2). Key observations include:High Accessibility Districts: Jing’an District, Changning District, Putuo District, Hongkou District, and Yangpu District exhibit higher average accessibility values. However, these districts also have higher standard deviation values, indicating greater imbalances in the spatial distribution of public sports facilities and their demand. Low Accessibility Districts: Jinshan, Songjiang, Qingpu, Fengxian, and Chongming districts show lower average accessibility values. Despite their lower standard deviations, which reflect relatively balanced spatial distributions, the overall accessibility in these districts is poor. Consequently, the spatial matching of supply and demand for public sports facilities in these areas is not well-aligned. In summary, public sports facilities in Shanghai exhibit significant disparities in spatial accessibility across both grid and street scales. High-accessibility areas are concentrated in central districts such as Jing’an, while peripheral districts like Chongming and Jinshan face challenges of low accessibility and poor spatial matching of supply and demand. These findings highlight the need for targeted improvements in the spatial layout of public sports facilities, particularly in low-accessibility areas.

**Table 2:** Spatial accessibility statistics of public sports facilities in Shanghai.

District name	Streets number	Grids number	Average of accessibility	Standard deviation of accessibility
Jing’an	14	40	0.52	0.16
Hong’kou	8	23	0.44	0.15
Huang’pu	10	19	0.42	0.11
Pu’tuo	9	57	0.41	0.16
Chang’ning	10	35	0.38	0.16
Xu’hui	14	55	0.35	0.13
Yang’pu	12	62	0.32	0.13
Bao’shan	13	317	0.21	0.16
Pu’dong	39	1460	0.14	0.10
Min’hang	13	373	0.12	0.06
Jia’ding	12	490	0.09	0.07
Song’jiang	16	604	0.07	0.05
Qing’pu	11	721	0.05	0.03
Feng’xian	12	739	0.04	0.02
Chong’ming	21	1676	0.02	0.02
Jin’shan	11	664	0.02	0.01

**Fig 12:**
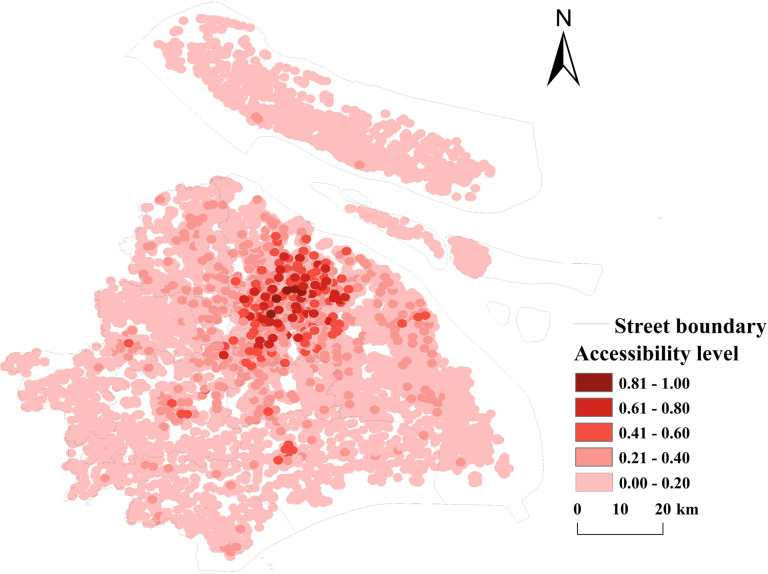
Results of accessibility of public sports facilities in Shanghai (buffer zone method) (Reprinted from OpenStreetMap under a CC BY license, with permission from OpenStreetMap. https://www.openstreetmap.org/copyright/zh-TW).

**(4) Sensitivity analysis of spatial accessibility:** In facility accessibility analysis, the Gaussian two-step moving search method provides a more detailed modeling of the decreasing service level with distance by introducing a distance decay function.However, this method is somewhat reliant on parameters, such as bandwidth and the choice of decay function. Therefore, it is necessary to use a sensitivity analysis to evaluate the robustness of its results.“To this end, the buffer zone method is employed as a comparative analysis tool in this study (see Fig 12). The buffer zone method is a straightforward and intuitive spatial analysis technique that assesses a facility’s service level based on the population coverage or density within a fixed service radius, offering a comparative reference for the Gaussian method.

①Consistency analysis: Through a visual comparative analysis of Figs 11 and 12, both methods consistently identify the central urban areas, such as Jing’an District and Huangpu District, as regions of high accessibility, demonstrating superior facility service coverage and effectiveness in these areas. Regarding distribution trends, both methods reveal that accessibility decreases progressively from the central urban areas to the peripheral regions. This indicates a high level of consistency between the two methods in capturing the overall spatial distribution characteristics.

②Difference analysis: The analysis results of the buffer zone method are highly dependent on the setting of the service radius, whereas the Gaussian method relies more on the bandwidth parameter and the distance decay function. This leads to notable differences in the peripheral urban areas. the buffer zone method indicates lower accessibility in edge urban areas, such as Chongming and Jinshan districts. In contrast, the Gaussian method, by considering the distance decay effect, reveals a certain potential service coverage capacity in these peripheral regions. In terms of coverage extent, the Gaussian method demonstrates a smoother range of values in high-accessibility areas, while the buffer zone method highlights significantly high coverage values within the service radius but exhibits a steep decline at the fringe areas.

The comparative analysis of these two methods validates the robustness and reliability of accessibility analysis results for public sports facilities in Shanghai. In the central urban areas, both methods consistently reveal high service coverage and spatial equity. However, in the fringe areas, the buffer zone method emphasizes the presence of service gaps, while the Gaussian method better models the potential service coverage, reflecting the dynamic characteristics of facility layout.

### 3.3. Social performance analysis

This study characterizes the regional equality and social equity of public sports facilities in Shanghai using the Gini coefficient, service radius coverage, and per capita location entropy to evaluate their social performance. On this basis, to further explore the issue of social equity in public sports facilities, a questionnaire survey was conducted to collect data on the usage and satisfaction levels of different population groups. The aim is to thoroughly analyze the disparities in the extent to which various social groups benefit from public sports facilities.

#### 3.3.1. Regional equity perspective.

**Fig 13:**
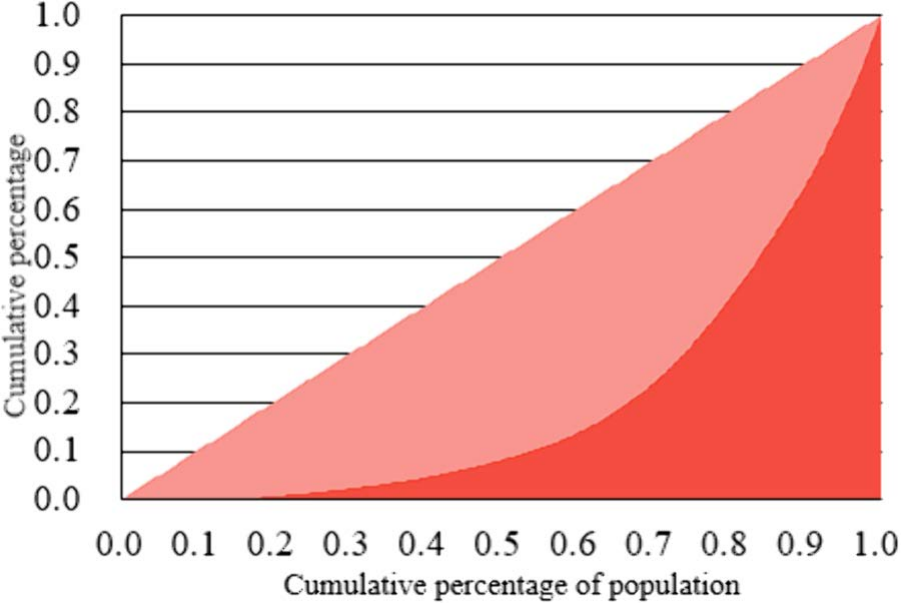
Lorenz curve for public sports facilities in Shanghai.

**(1) Gini coefficient and Lorenz curve:** The Gini coefficient of public sports facilities in Shanghai was calculated to be 0.58, which falls within the range of 0.4 to 0.59, indicating an excessive disparity in resource allocation and an unbalanced spatial distribution. Based on the Gini coefficient, all streets in the study area were ranked in ascending order according to public sports facilities per capita. Using 10% increments as nodes, the proportion of public sports facilities owned by each 10% segment of the resident population was measured, leading to the construction of the Lorenz Curve (see Fig 13).

**Fig 14:**
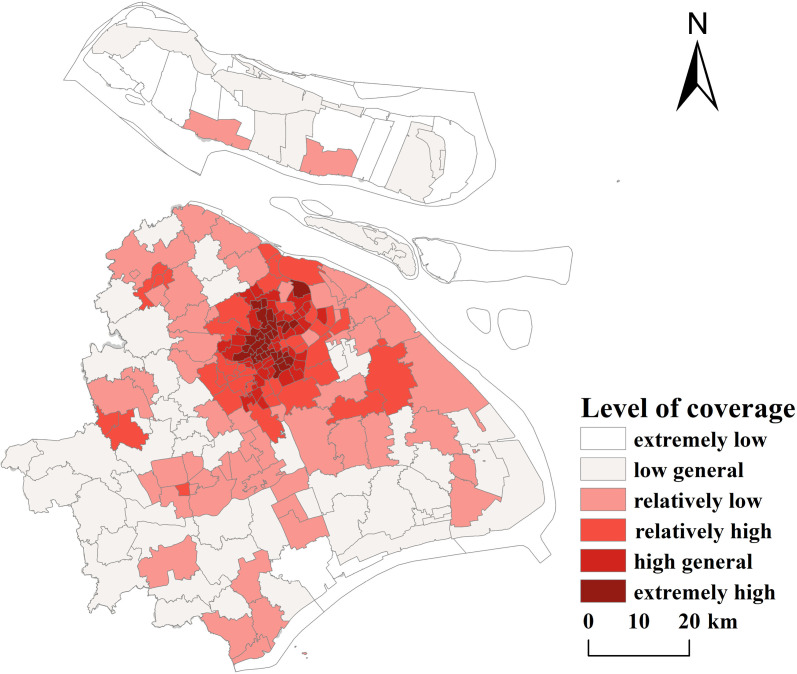
Level of coverage of public sports facilities in Shanghai (Reprinted from OpenStreetMap under a CC BY license, with permission from OpenStreetMap. https://www.openstreetmap.org/copyright/zh-TW).

**(2) Service radius coverage:** The coverage index of public sports facilities for each street in Shanghai was calculated, visualized, and classified into six hierarchical levels to further analyze the spatial differentiation of public sports facilities at the street level. Within the study area: 32 street units have extremely high public sports facilities coverage indexes, primarily concentrated in the central city, demonstrating significant clustering characteristics (see Fig 14). 89 street units with low coverage indexes are predominantly distributed in suburban areas, including Fengxian District, Chongming District, Jiading District, and Baoshan District. These findings align with the Gini coefficient results, further highlighting the uneven spatial distribution of public sports facilities in Shanghai. The spatial differentiation of the coverage index is also closely coupled with the spatial accessibility pattern.

#### 3.3.2. Social equity perspective.

**Fig 15:**
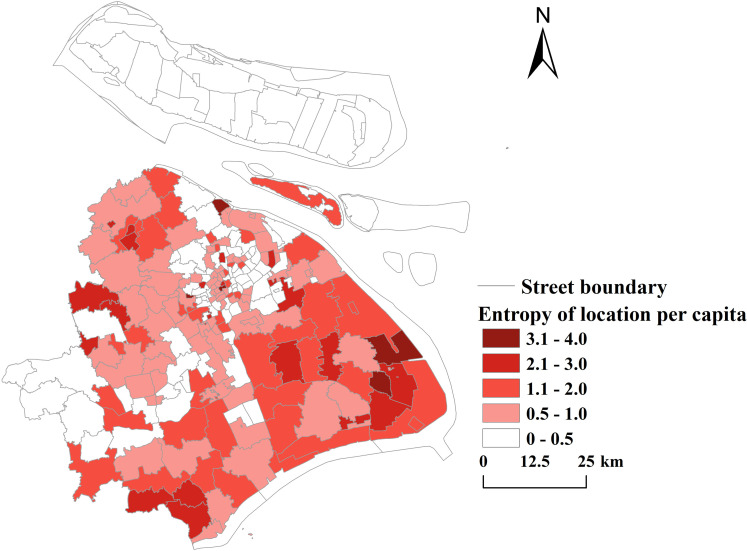
Evaluation of per capital location entropy in Shanghai (Reprinted from OpenStreetMap under a CC BY license, with permission from OpenStreetMap. https://www.openstreetmap.org/copyright/zh-TW).

The locational entropy value of the service capacity of public sports facilities in each spatial unit was attained by calculation (see Fig 15). The spatial distribution of the locational entropy of public sports facilities in Shanghai generally exhibits the characteristic of being high in the east and the west and low in the middle. The highest locational entropy value is 4.25, observed in Youyi Road Street in Baoshan District, while the lowest value is 0.02, found in the Caohejing Emerging Technology Development Zone, also in Baoshan District. Out of the 225 streets within the study area, only 67 streets have locational entropy values greater than 1, accounting for 29% of the total. This indicates that the service level of most public sports facilities in the study area is below the average level, and the locational entropy values vary significantly across spatial units.

The results of the questionnaire survey reveal that while public sports facilities bring numerous benefits to people across different age groups, various challenges persist. Overall, in the older urban areas, the existing facilities, though established, are outdated and slow to be updated. This creates a noticeable gap between the needs of young people and the current state of public sports facilities. In suburban areas, the construction of public sports facilities remains insufficient, with facilities in large parks distributed sporadically. Moreover, spatial constraints in older neighborhoods lead to confined fitness spaces. Approximately 60% of the residents aged 18–30 reported that long travel times to public sports facilities significantly limit their frequency of use. For older adults aged over 60, about 70% noted that the distance to facilities, coupled with travel inconveniences, restricts them to visiting public sports facilities fewer than three times a week for exercise. In Chongming District, which is the least accessible area in Shanghai, more than 80% of elderly residents reported a severe shortage of fitness equipment in their communities, with an average of only eight pieces of equipment per 50 elderly individuals. Furthermore, over 90% of these pieces of equipment has been in use for more than five years, exhibiting significant wear and tear and presenting safety hazards. Conversely, in the central urban areas, despite the abundance of sports facilities, there are issues with the structure and distribution of facility types. Jing’an District has the highest accessibility in Shanghai, such as Caojiadu Street, which is located in Jing’an District, had the accessibility of 0.8775, but the distribution of facilities within the district is relatively uneven. There are as many as 711 facilities as “Citizen Puzzle Fitness Court”, which tend to focus on meeting the needs of the elderly exercise, on the other hand, there are only 56 “Citizens’ Courts”, which are in short supply for young people.

In summary, the uneven distribution, remoteness and poor quality of facilities have hindered residents’ convenient and effective use of public sports facilities. This underscores the inadequate consideration of age-specific needs in the allocation of social resources which undermines social equity. Furthermore, it reflects suboptimal social performance requiring urgent enhancement and optimization.

#### 3.3.3. Sensitivity analysis of social performance.

To further verify the reliability and robustness of the aforementioned social performance analysis results, this study conducts a sensitivity analysis of the outcomes derived from the three methods. Specifically, the study evaluates the consistency and discrepancies between high and low performance regions and examines the complementarity of each method in capturing distinct spatial characteristics (see Table 3).

**Table 3:** Multi-method comparison table for social performance analysis of Shanghai public sports facilities.

Method	High-performing regions	Low-performing regions	Overall trend	Characteristics
Gini coefficient	Jing’an District, Changning District, etc.	Jinshan District, Chongming District, etc.	Resource distribution gap is large, higher in the center and lower in the peripheral areas	Suitable for overall fairness evaluation
Service radius coverage	Jing’an District, Changning District, etc	Jinshan District, Chongming District, etc.	Higher coverage in the center, lower coverage in the periphery	Intuitively reflecting the coverage of the facilities
Per capita service location entropy	Jing’an District, Huangpu District, etc. distribution centers	Qingpu District, Chongming District, etc. direction	Higher equilibrium in the center, poorer service in the peripheral areas	Focus on per capita service level

①Consistency Analysis:In terms of overall resource distribution fairness (measured by the Gini coefficient), facility coverage level (service radius coverage), and per capita service balance (per capita service location entropy), central urban areas such as Jing’an and Huangpu districts consistently emerge as regions with higher social performance. The consistency results across the three methods also indicate that peripheral areas, such as Chongming and Jinshan districts, face significant resource scarcity, limited service coverage, and lower per capita facility service levels.
②Difference Analysis:The Gini coefficient emphasizes the overall fairness of facility resource distribution but cannot capture the level of facility coverage in specific regions. In contrast, service radius coverage focuses on the extent to which facilities serve residents, providing an intuitive and specific reflection of accessibility and service scope. However, it does not fully account for variations in population density. Lastly, the entropy of per capita service location highlights the balance between population and facilities, effectively reflecting the fairness of facility services. In summary, the social performance analysis of public sports facilities in Shanghai demonstrates strong robustness.

## 4. Discussion

### 4.1. Analysis of the current situation

**(1) Spatial Configuration of Public Sports Facilities:** The spatial layout of public sports facilities is shaped by the long-term interplay of various factors such as natural conditions, socio-economic characteristics, and historical development [54]. Analyzing the clustering characteristics and accessibility of public sports facilities in Shanghai reveals the need for a more uniform configuration. The clustering pattern is characterized by “central agglomeration + peripheral clusters,” where streets with high accessibility are concentrated in the central urban areas and form a checkerboard-like mosaic in the surrounding regions. Several factors contribute to this spatial configuration:

①Land Price: Land prices play a significant role in explaining the heterogeneity in the spatial supply-demand relationship of public sports facilities. High land price areas typically feature strong consumer demand, leading to a concentrated distribution of facilities with higher levels of accessibility.
②Population: Traditionally, the main urban districts, such as Huangpu, Xuhui, Jing’an, Changning, Putuo, and Hongkou, are characterized by higher levels of urbanization, earlier construction years, dense populations, and commercial activity. These factors contribute to a better supply-demand match and higher accessibility in central urban areas. In contrast, peripheral urban areas exhibit poorer matching relationships, with lower accessibility.
③Transportation: Transportation infrastructure, including road network density and the number of bus stops, significantly affects the accessibility of public sports facilities. A denser road network correlates with higher accessibility, as it facilitates easier access to facilities. For example, in the Pudong New Area, despite its large area and significant population, public sports facilities remain relatively scarce compared to the city center. The coverage of the 10-minute walking fitness circle is low, leaving many streets in a state of “high demand – low supply.”(2) Analysis of the current situation of social performance.

To address these issues, Shanghai must actively promote incremental construction, eliminate service blind zones, and enhance the supply and equitable distribution of public sports facilities across municipal districts. This will improve the spatial fairness and accessibility of public sports facilities in the city.

**(2) Social Performance of Public Sports Facilities:** Building on the spatial allocation analysis, the social performance of Shanghai’s public sports facilities is evaluated from three perspectives: the Gini coefficient, service radius coverage, and per capita service area entropy.

①Gini Coefficient: The Gini coefficient, an essential measure of resource distribution fairness, is calculated at 0.58 for Shanghai’s public sports facilities. This reflects a significant inequality in resource allocation, with facilities concentrated in the central urban areas and exhibiting high accessibility, while peripheral urban areas face a severe shortage. Such uneven distribution not only hinders social equity but also limits residents in peripheral urban areas from enjoying services comparable to those in central regions. To reduce this disparity, it is crucial to increase investment in peripheral urban areas, narrow the inter-regional gap, and enhance overall social equity.
②Service Radius Coverage: Service radius coverage assesses the extent to which public sports facilities effectively serve nearby residents. In Shanghai, the 10-minute walking fitness circle is well-established in central urban areas due to the high density of facilities, enabling convenient access for residents. However, peripheral urban areas exhibit lower coverage, with many zones classified as service blind spots. This uneven coverage limits the ability of residents in peripheral regions to access facilities for daily fitness activities, negatively impacting their health and quality of life. Addressing this issue requires systematic planning and incremental facility construction to ensure adequate coverage for all regions.
③Per Capita Service Area Entropy: Per capita service area entropy measures disparities in the level of service across different regions. Central urban areas exhibit higher entropy, reflecting better facility utilization and higher service levels. In contrast, peripheral urban areas show lower entropy due to insufficient facilities and a mismatch with residents’ demands. This disparity arises from the uneven distribution of resources: facilities are heavily concentrated in high land price areas, while peripheral regions lack sufficient supply. To improve per capita service entropy in peripheral areas, it is necessary to optimize facility layout, increase the number of facilities, and ensure alignment with residents’ demands, thereby enhancing overall utilization efficiency.

The analysis reveals a stark imbalance in the social performance of Shanghai’s public sports facilities. Central urban areas benefit from dense facility distribution, high accessibility, and high utilization efficiency, whereas peripheral urban areas suffer from insufficient supply, low service coverage, and low utilization efficiency. These disparities result in poor overall social performance. To achieve balanced development and improve social performance, greater investment is needed in peripheral urban areas. This includes optimizing the layout of facilities, improving their quality, and enhancing service levels. Such measures will promote social equity and enhance the health and quality of life for all residents [55,56].

### 4.2. Configuration optimisation suggestions

**(1) Building a Hierarchical and Balanced Spatial Layout for Urban Public Sports Facilities:** The centroid model is a commonly used method for optimally integrating public sports facilities into a hierarchical and balanced spatial layout. By leveraging the radiation and aggregation effects of large sports venues, multiple sports centers can be developed in urban areas. This involves the organic combination of large sports venues with smaller public sports facilities to construct a spatial layout model that meets the needs of urban residents [57].Public sports facilities can be categorized into three levels—advanced, intermediate, and primary—based on the varying needs of urban residents, with each level corresponding to a distinct type of sports center. Advanced sports centers are fewer in number, cover larger areas, have extensive radial ranges, and offer diverse sports activities. In contrast, primary sports centers occupy smaller areas and are typically focused on fewer and more specific types of sports.When integrating and optimizing sports centers, careful consideration must be given to facility accessibility and the effective combination of spaces of different scales and types. Therefore, the layout of urban public sports facilities should ensure a scientifically sound and balanced distribution of advanced, intermediate, and primary facilities to meet diverse urban demands.

**(2) Driving Functional Upgrades of Public Sports Facilities Through Digital Transformation:** The rapid development and widespread application of digital and information technologies in the sports field have created significant opportunities for upgrading the functionality of public sports facilities. As critical platforms for hosting sports events, athletic training, and public fitness, public sports facilities can better meet the demands of modern development through digital transformation. The construction of smart sports venues should become a key direction for future development. By leveraging emerging technologies such as the Internet of Things (IoT), big data analytics, and artificial intelligence (AI), the functionality and operational efficiency of sports facilities can be significantly enhanced. Shanghai should seize the opportunities presented by digital development to upgrade the functionality of its public sports facilities. Through the integration of new technologies, the city can drive the transformation and modernization of its sports infrastructure, aligning it with the needs of a rapidly evolving urban environment.

**(3) Maximizing Urban Space Potential: Enhancing Targeted Supply Capacity:** To maximize the potential of urban spaces, particularly underutilized areas referred to as “golden corners and silver edges,” this study proposes an innovative approach that integrates public sports facilities with various urban domains. The strategy involves using “lost spaces” as an entry point, guided by a “targeted acupuncture” redevelopment concept, a “participatory” decision-making logic, and a “gradual” renewal strategy. Various forms of spatial revitalization are implemented, such as activating under-bridge spaces, utilizing rooftop spaces, regenerating waterfront areas, embedding parks and green spaces, and rehabilitating irregular spaces. These efforts aim to strengthen the integration of sports facilities with green spaces, transportation, education, culture, and water resources. The following are representative case studies of this approach: ①”Sports + Green Spaces”: The Taopu Central Green Park in Shanghai serves as an example of combining green spaces with underground sports facilities. The project occupies approximately 30,000 square meters and includes ball game halls, swimming pools, skating rinks, and supporting sports facilities, exemplifying the integration of ecological and recreational spaces. ② “Sports + Transportation”: The sports facilities beneath the Middle Ring Road in Putuo District, Shanghai, demonstrate the effective utilization of under-bridge spaces. Located south of Yunling Road and within the Middle Ring Road near Zhenbei Road, the project covers an area of approximately 15,000 square meters and features outdoor sports facilities such as basketball courts and extreme sports areas. ③ “Sports + Water Resources”: The West Outer Ring River Water Sports Center in Changning District, Shanghai, showcases the integration of water resources with sports facilities. The project combines new docks with inland waterways to create water sports infrastructure, providing residents with access to aquatic activities while optimizing the use of water resources.

(4) **Implementing a Precise and Demand-Oriented Mechanism for the Dynamic Allocation of Sports Facilities:** First, identify demand differences and optimize resource allocation. Big data analytics can be leveraged to assess the diverse sports needs of different regions and age groups. For instance, young residents in emerging urban areas may require ballparks and fitness facilities, while elderly populations in aging communities may prefer rehabilitation and soothing equipment. Based on these insights, priority will be given to allocating funds and land to areas with the most urgent needs, minimizing resource mismatches, and enhancing alignment with residents’ requirements. Second, establish a dynamic deployment process to improve time management. A cross-departmental team will be formed to flexibly manage the utilization of facilities based on data such as usage duration, frequency, and peak time slots. This approach ensures the efficient and responsive use of public sports resources.

### 4.3. Inadequacies

This study still has several limitations: ①Data on Public Sports Facilities: First, in terms of data coverage, only public sports facilities listed on official government websites are included. However, facilities such as enterprise-built stadiums are not fully accounted for, making it difficult to represent the actual availability of all sports facilities. Second, the timeliness of the data is limited. Although the dataset is current up to 2023, it cannot fully reflect the dynamic changes in the construction, renovation, or demolition of sports facilities, which may affect the accuracy of the analysis.② Limitations in Travel Mode Analysis: This study considers walking as the sole daily travel mode for residents and does not account for the impact of other travel modes, such as public transportation or driving on spatial accessibility. Different travel modes lead to varying travel times, directly influencing the public’s choice of sports facilities. Therefore, the effect of diverse travel modes on the spatial accessibility of public sports facilities requires further exploration.③ Socio-Economic Attributes: The study does not incorporate the influence of socio-economic attributes on spatial accessibility, and its scope is primarily limited to public sports facilities. Future research should integrate socio-economic factors, such as income level, population density, or education, to provide a more comprehensive analysis.④ Complexity of Spatial Differentiation: Public sports facilities exhibit significant spatial differentiation in terms of supply, type, and demand. The increasingly individualized fitness needs of residents have created intense competition among different types of fitness venues. In this context, developing a spatial accessibility evaluation framework that accounts for the heterogeneity of supply-and-demand relationships while balancing diverse and personalized fitness needs remains a challenging task. Special attention should also be given to vulnerable groups, such as youth and the elderly, to ensure equitable access to fitness resources.

## 5. Conclusion

This study analyzes the spatial distribution characteristics, accessibility, and social performance of public sports facilities in Shanghai from both grid and street scales, as well as from the perspectives of supply and demand, spatial equilibrium, and other dimensions. The findings are as follows.

(1) Spatial Distribution and Accessibility: The spatial distribution of public sports facilities in Shanghai exhibits a pattern of central urban agglomeration and peripheral urban scattering, with a significant directional coupling along the northeast-southwest population distribution. At the grid scale, accessibility is higher in central areas and lower in peripheral regions. At the street scale, accessibility is highest in Jing’an District and lowest in Pudong New District, although the central area demonstrates a notable imbalance between supply and demand. Overall, the spatial match between supply and demand remains limited, showing clear geographical disparities.
(2) Resource Allocation and Social Equity: Public sports facilities in Shanghai are unevenly distributed, with a Gini coefficient as high as 0.58, indicating a pronounced spatial imbalance. At the street level, the central urban area contains a disproportionately high share of facilities, while suburban areas experience a relative lack of resources. This results in uneven coverage within the service radius, characterized by agglomeration in the central city and insufficient coverage in the suburbs. From a social equity perspective, service capacity is higher in the eastern and western areas but lower in the central regions, with most streets falling below the average service level.
(3) Accessibility-Equity Interrelationships: At both street and grid scales, accessibility and equity exhibit a complex interrelated distribution. Specifically, streets with higher accessibility for certain types of sports facilities also perform better in terms of equity, as indicated by higher coverage indices. This inter-coupling suggests that increased accessibility on a given street contributes to a more balanced distribution of public resources, thereby improving equity at the spatial level. The observed correlation reveals a nuanced relationship between accessibility and equity, underscoring the dynamic interplay between these two dimensions in the spatial distribution of public sports facilities.

The findings of this study hold significant implications for urban planning and public health in Shanghai. In future urban planning efforts, the development of peripheral urban areas, such as Chongming and Jinshan districts, should prioritize the supplementation of public sports facilities to accommodate population growth and distribution. Planning for sports facilities should fully consider the coupling relationship between population distribution and sports facilities across different regions, as well as trends in population growth and demographic changes. For regions with rapid population growth and a higher proportion of young residents, facilities catering to the needs of younger groups, such as ballparks and fitness gyms, should be added. Conversely, regions with a large elderly population should see the addition of rehabilitative and soothing sports facilities tailored to the needs of older adults. From a public health perspective, the imbalance between the supply and demand of sports facilities in central urban areas may result in uneven fitness resource distribution among residents, which is detrimental to public health outcomes. Future research should focus on improving residents’ fitness conditions by optimizing the spatial layout of facilities and adjusting the proportions of different facility types. These measures can, in turn, enhance overall public health outcomes. In line with national policies advocating the integration of physical activity and healthcare, the planning of public sports facilities should actively respond by emphasizing coordinated layouts with nearby medical institutions. Additionally, a digital information interaction platform should be established to dismantle data barriers between the sports and medical sectors, thereby facilitating the high-quality development of public health services.

## Supporting information

S1 File Shanghai_Township boundary. (DBF)

S2 File Shanghai_Township boundary. (PRJ)

S3 File Shanghai_Township boundary. (SBN)

S4 File Shanghai_Township boundary. (SBX)

S5 File Shanghai_Township boundary. (SHP)

S6 File Shanghai_Township boundary. (SHX)

S7 File Shanghai_Township boundary. (XML)

S8 File Shanghai public sports facilities basic datas. (XLSX)

S9 File Shanghai_chn_ppp_2020_UNadj. (OVR)

S10 File Shanghai_chn_ppp_2020_UNadj. (TIF)

S11 File Shanghai_chn_ppp_2020_UNadj. (XML)

S12 File Shanghai_chn_ppp_2020_UNadj. (OVR)

S13 File Shanghai_chn_ppp_2020_UNadj. (XML)
